# A multicenter, retrospective analysis of elderly patients with acute myeloid leukemia who were treated with decitabine

**DOI:** 10.18632/oncotarget.23823

**Published:** 2018-01-02

**Authors:** Jun Ho Yi, Silvia Park, Jung Han Kim, Young-Woong Won, Do Hyoung Lim, Boram Han, Jieun Uhm, Hae Su Kim, Chul Won Jung, Jun Ho Jang

**Affiliations:** ^1^ Division of Hematology-Oncology, Department of Medicine, Chung-Ang University Hospital, Seoul, Korea; ^2^ Division of Hematology-Oncology, Department of Medicine, Samsung Medical Center, Sungkyunkwan University School of Medicine, Seoul, Korea; ^3^ Department of Internal Medicine, Kangnam Sacred-Heart Hospital, Hallym University Medical Center, Hallym University College of Medicine, Seoul, Korea; ^4^ Division of Hematology and Oncology, Department of Internal Medicine, Hanyang University Guri Hospital, Hanyang University College of Medicine, Guri, Korea; ^5^ Department of Internal Medicine, Dankook University College of Medicine, Cheonan, Korea; ^6^ Department of Internal Medicine, Hallym University Medical Center, Hallym University, Hallym University College of Medicine, Anyang, Korea; ^7^ Division of Hematology and Oncology, Department of Internal Medicine, Hanyang University Seoul Hospital, Hanyang University College of Medicine, Seoul, Korea; ^8^ Division of Hematology-Oncology, Department of Internal Medicine, VHS Medical Center, Seoul, Korea

**Keywords:** acute myeloid leukemia, elderly patients, decitabine

## Abstract

Decitabine is widely accepted as the treatment options for elderly acute myeloid leukemia (AML) patients. However, the efficacy has yet been assessed in Asian population. We retrospectively analyzed the outcomes of 80 Korean elderly AML patients who were treated with decitabine. The median age was 74 years (range, 64 to 86 years) and 6 (7.5%), 48 (60.0%), and 25 (31.3%) patients were categorized to favorable, intermediate, and poor risk group, respectively. The median OS was 10.2 months (95% CI 5.0–15.4). Given that decitabine treatment demonstrated improved clinical outcomes, it could be considered as one of the first-line treatment for Korean elderly AML patients.

## INTRODUCTION

Acute myeloid leukemia (AML) is the most common type of leukemia in adults, with more than 12,000 people are diagnosed annually in the United States [1]. Basically, AML is a disease of the elderly as the median age of the newly diagnosed patients is over 65 years [2]. However, the treatment options for the elderly AML patients have been limited, and they usually show poor clinical outcomes owing to their poor performance, comorbidity, unfavorable cytogenetics, or prior hematologic neoplasms. Thus, age per se is considered as one of the major prognostic factors. Although several studies have suggested that elderly AML patients still benefit from intensive chemotherapy [3], a substantial portion of the patients are not suitable for intensive treatment, and there have been unmet needs for this large population.

While various pathways are known to be involved in the development of myeloid neoplasms, recently, epigenetic changes have been turned out to play important roles in leukemogenesis [4]. Decitabine (5-aza-2′-deoxycytidine, Dacogen^®^) which inhibits DNA methyltransferase, demonstrated promising results in patients with high-risk myelodysplastic syndrome [5, 6]. With these results, two prospective trials in elderly AML patients had been conducted which showed improved clinical outcomes and acceptable safety profiles compared to conventional treatments [7, 8]. Based on these data, the use of decitabine in elderly AML patients is approved by the European Committee.

Meanwhile, the superiority of decitabine over conventional treatment was not widely accepted as US FDA rejected its use in elderly AML patients. Furthermore the efficacy or safety of this agent has not been evaluated in Asian population where difference of clinical manifestation or cytogenetics had been noted [9, 10]. In the current study, we conducted a multicenter, retrospective analysis on elderly AML patients from 8 tertiary institutes in Korea who were treated with decitabine in order to confirm whether the clinical outcomes of this agent are also acceptable in this population, and to provide further understanding of the disease nature of AML arisen in elderly patients.

## MATERIALS AND METHODS

### Patients

Patients diagnosed with AML from 2013 to 2016 were included in the analysis. The inclusion criteria were as follows: (1) 65 or older patients with newly diagnosed, histologically confirmed AML (myeloid blast ≥ 20% either in the bone marrow or peripheral blood); (2) Treated with decitabine in a schedule of 20 mg/m^2^ for five days every 4 weeks in patients with adequate organ functions determined by physicians in charge. Of note, patients with acute promyelocytic leukemia, central nervous involvement, or other active systemic malignancies were excluded.

Prior anti-leukemic treatments were not allowed other than cytoreductive hydroxyurea. Clinical parameters including age, sex, performance status, cytogenetic [11]/molecular [12] risk group, baseline laboratory findings, treatment outcomes were collected retrospectively from patients’ medical records. Follow-up bone marrow biopsies and aspirations were performed at the discretion of the attending physicians. Prophylactic antibiotics, antifungals, or antivirals were administered following the standard protocols of the institutes. The institutional review boards of all participating centers approved the study.

### Statistical analysis

The primary end-point of the study was OS which was measured from the date of diagnosis of an AML to the date of death or last follow-up using the Kaplan-Meier method. OS was compared using a log-rank test. Secondary end-point was complete response rate. *P*-values < 0.05 were considered statistically significant, and all *P-*values corresponded to two-sided significance tests. Statistical analysis was performed using SPSS software version 17.0.

## RESULTS

### Patient characteristics

A total of 80 patients were eligible for the analysis. The median age of patients was 74 years (range, 64 to 86 years) and 49 patients (61.3%) were male. All patients were Korean. Regarding the risk group, 6 (7.5%), 49 (61.2%), and 25 (31.3%) cases were classified as favorable, intermediate, and poor risk group, respectively. None of the patients had undergone hematopoietic stem cell transplantation. Other details including performance scale and baseline laboratory findings are described in Table 1.

**Table 1:** Baseline characteristics of the patients (*N* = 80)

Characteristics	*N* (%)
Age, years	
Median (range)	74 (65–86)
70 or younger	22 (27.5)
Older than 70	58 (72.5)
Sex	
Male	49 (61.2)
Female	31 (38.8)
Risk group	
Favorable	6 (7.5)
Intermediate	48 (60.0)
Poor	25 (31.3)
Unknown	1 (1.2)
ECOG^*^ performance scale	
0	1 (1.3)
1	11 (13.8)
2	31 (38.8)
3	31 (38.8)
4	6 (7.5)
Type of AML	
* De novo*	43 (53.7)
Secondary	22 (27.5)
Unknown	15 (18.8)
Bone marrow blast percentage	
Median (range)	45.7 (14.0–99.0)
Peripheral blood blast percentage	
Median (range)	41.0 (0.0–96.0)
White blood cells (/㎕L)	
Median (range)	11,000 (200–253,910)
Hemoglobin (g/dL)	
Median (range)	8.1 (3.2–16.9)
Platelet (/㎕L)	
Median (range)	56,000 (3,000–890,000)

^*^ECOG, Eastern Cooperative Oncology Group.

### Clinical outcomes of decitabine treatment

With the median follow-up duration being 19.6 months (95% confidence interval (CI) 15.2–24.0), the patients had received median 3 (range 1–27) cycles of treatment. Among 80 patients, only 35 (43.8%) had been evaluated bone marrow response, and complete remission was noted in 10 patients. The major reasons of not undergoing bone marrow evaluation were inadequate hematologic response of peripheral blood and poor performance status.

**Figure 1:**
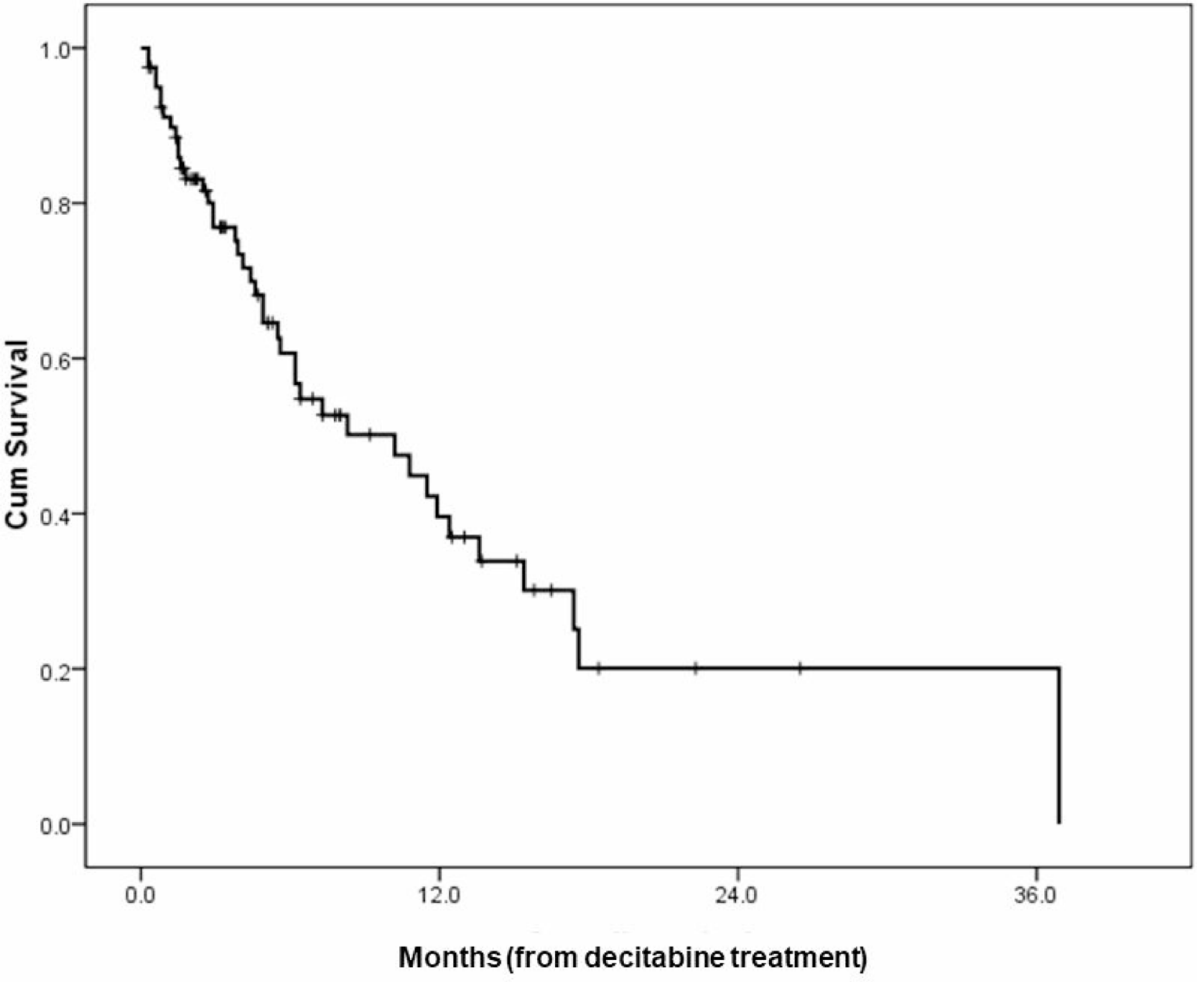
An overall survival curve of all patients

**Figure 2:**
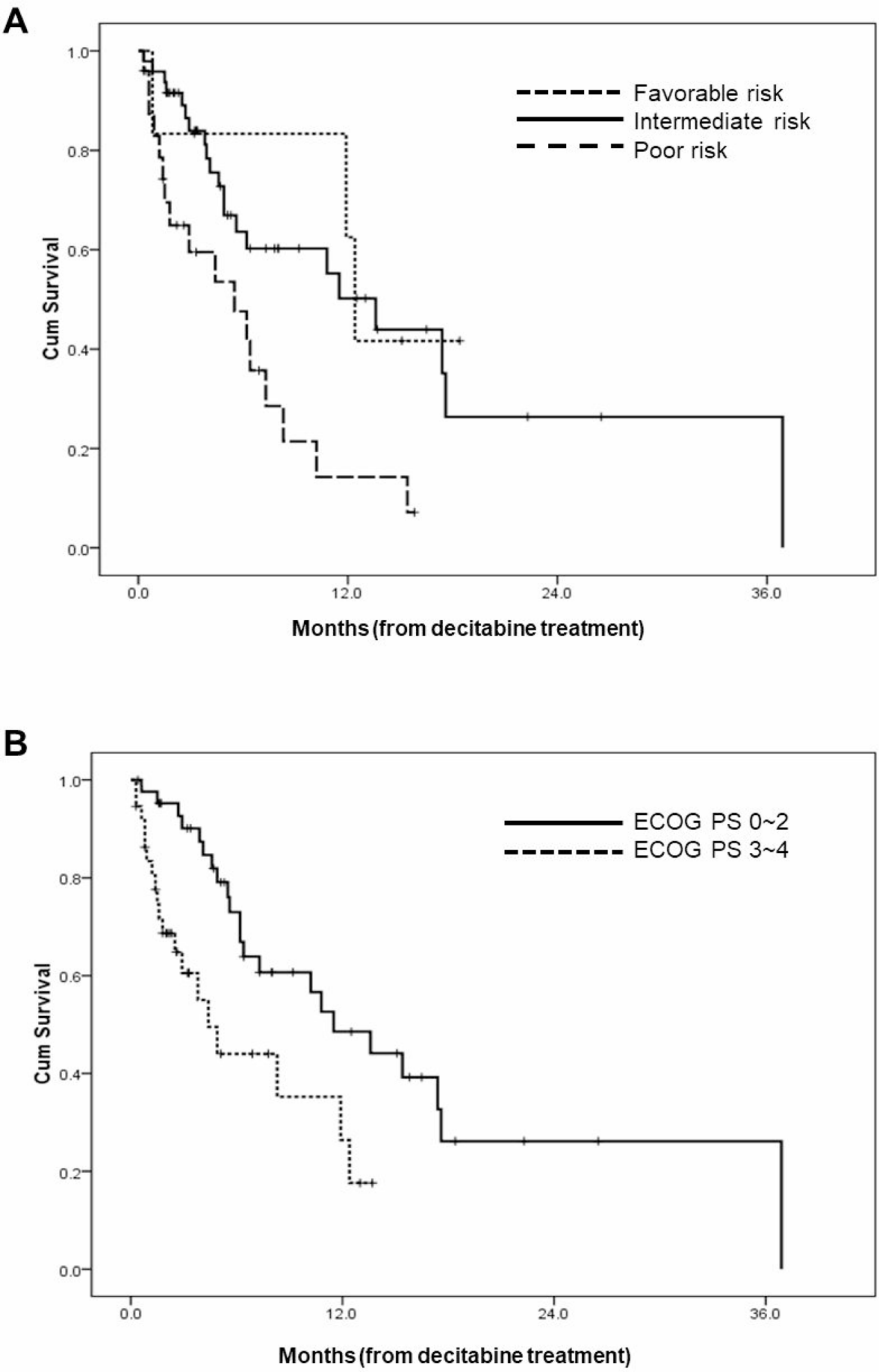
Overall survival curves according to (**A**) cytogenetic/molecular risk groups; (**B**) performance scales.

The median OS for all patients was 10.2 months (95% CI 5.0–15.4) (Figure 1), and the 1-year survival rate was 38.3%. The median OS durations according to the cytogenetic risk group are as follows; 12.4 months (95% CI 11.4–13.4) for favorable risk group (*N* = 6), 13.6 months (95% CI 8.7–18.5) for intermediate risk group (*N* = 49), and 5.5 months (95% CI 1.4–9.6) for poor risk group (*N* = 25) (*p* = .001). Another prognostic factor was Eastern Cooperative Oncology Group performance scale (ECOG-PS). When we categorized our cohort into two groups, that is ECOG-PS 0∼2 *vs.* ECOG-PS 3 & 4, those with good performance status demonstrated improved survival (11.5 months (95% CI 6.6–16.4) *vs.* 4.4 months (95% CI 2.4–6.4), *p* = .004). The OS curves according to prognostic factors are provided in Figure 2. Other clinical factors such as age, sex, bone marrow blast percentage, or white blood cell count did not discriminate patients’ outcome (Table 2).

**Table 2:** Univariate analysis for overall survival (months)

	*N*	Overall survival (95% CI)	*p*
Age			.963
70 or younger	22	10.8 (2.0–19.6)	
Older than 70	58	8.3 (2.7–13.9)	
Sex			.396
Male	49	6.2 (0.5–11.9)	
Female	31	15.4 (5.9–24.9)	
Risk group (*N* = 79)			.006
Favorable	6	12.4 (11.4–13.4)	
Intermediate	48	13.6 (8.7–18.5)	
Poor	25	5.5 (1.4–9.6)	
ECOG^*^ performance scale			.004
0∼2	43	11.5 (6.6–16.4)	
3∼4	37	4.4 (2.4–6.4)	
Bone marrow blast percentage (*N* = 77)			.652
20∼30%	28	6.4 (3.9–8.9)	
31 or higher %	49	10.2 (3.4–16.8)	
White blood cells (/㎕L)			.536
∼20,000	50	6.4 (0.7–12.1)	
20,001 ∼	30	11.5 (5.8–17.2)	

In terms of infectious adverse events, 13 (29.5%) out of 44 patients who had received at least 3 cycles of treatment have experienced at least 1 infectious adverse events during the first 100 days of treatment. Eight patients had bacterial infections, 3 had fungal infections, and 2 had infections with multiple etiologies.

## DISCUSSION

In the present study, we retrospectively analyzed 80 elderly patients with AML who were treated with decitabine at a schedule of 20 mg/m^2^ for 5 days every 4 weeks. Decitabine was well tolerated, and patients received a median of 3 cycles of treatment with the median OS being 10.2 months. The subset analyses have shown that cytogenetics and ECOG-PS were prognostic factors. About one thirds of patients had experienced infectious adverse events during the first 100 days.

AML is the most common form of acute leukemia in adults. While the 5-year survival rate for patients less than 65 years was 39.5%, that of patients over 65 years was 5.2% [13]. Intensive chemotherapy can be applied for selected patients with good performance status and favorable cytogenetics, which may result in prolonged survival [14]. For patients who are unlikely to benefit from the intensive treatment, several less-intensive treatment options have been introduced, such as hydroxyurea or low-dose cytarabine (LDAC). Recently, two hypomethylating agents have been introduced in the treatment of elderly AML patients [8, 15, 16], and among them, in Korea, decitabine is the only agent that was approved and reimbursed for elderly AML patients.

While various doses and schedules have been evaluated, the efficacy and safety of current standard dose and schedule, that is, 20 mg/m^2^ daily for 5 days every 4 weeks, were evaluated in two studies. In a phase II study of Cashen *et al.*, 55 patients with AML older than 60 years were treated with decitabine. The overall response rate was 25% and the median overall survival was 7.7 months with the 30-day mortality rate being 7% [7]. A subsequent phase III trial named DACO-106 was carried out in 485 patients with AML of intermediate or poor risk cytogenetics older than 65 years and they were randomized to receive either decitabine or physician’s choice (best supportive care, BSC or LDAC) [8]. With the median age of the patients being 73 years, decitabine was associated with non-significant but favorable trend of increased OS (7.7 months *vs.* 5.0 months, *p* = .108, hazard ratio 0.85 (95% CI 0.69–1.04)), and an unplanned analysis performed after 1 year demonstrated the significant survival benefit (7.7 months *vs.* 5.0 months, *p* = .0.37, hazard ratio 0.82 (95% CI 0.68–0.99)). Remission rate was also higher in decitabine arm, as it was associated with a significantly higher rate (17.8% *vs.* 7.8%, respectively, *p* = .001). However, there were several criticisms regarding the results. First, significant survival benefit was demonstrated only after additional, unplanned analysis. Second, the clinical outcomes with LDAC were unsatisfactory compared to prior studies. Third, there was a regional variation in survival as the majority of benefit was noted in confined to Eastern European patients. And one of the reasons we carried out the current analysis was because just small number of Asian patients were included in the DACO-016 trial, it was necessary to assess the efficacy of decitabine in Asian elderly patients.

The decision of the front-line treatment for elderly AML patients is a challenging issue. To evaluate the efficacy of decitabine compared to BSC or intensive chemotherapy in elderly AML patients, we compared our data to the data of a study by Kim *et al.* [17], in which retrospective analyses of 477 Korean elderly AML patients who were treated with either BSC or intensive chemotherapy were undertaken (Table 3). In terms of patient characteristics, our cohort is consisted of older patients, and the proportion of poor performance/cytogenetics was higher in our cohort. The comparison of survival data, however, demonstrated interesting findings. The OSs of BSC group, our cohort, and intensive chemotherapy group were 86 days (95% CI 54–118), 286.6 days (140–431.2), and 339 days (275–403), respectively. Although it is not a head-to-head comparison, it seems that OS of our cohort is quiet better than that of BSC group. While the numerical data are superior in intensive chemotherapy group, their younger age and better performance status may have contributed to it. Subset analyses showed similar findings. Compared to the BSC group, the survival outcomes of our cohort are better, in the same age, performance scale, and cytogenetic risk groups. Compared to intensive chemotherapy group, the numerical data are slightly inferior in many fields but the difference is not a big number and besides, in a few fields like age over 76 years or ECOS-PS 3, OS was superior in our cohort. Although it is difficult to make a firm conclusion with these results, our data may imply that decitabine treatment should be considered for these patients. Further investigations, including prospective clinical trials are required.

**Table 3:** Comparison of the Korean retrospective analysis and the current study

	Best supportive care (*N* = 211)	The current study (*N* = 80)	Intensive chemotherapy (*N* = 266)
Age (median, range)	72 (60–101)	74 (64–86)	66 (60–86)
Age > 70 years (*N*, %)	126 (59.7)	58 (72.5)	52 (19.5)
Performance (*N*, %)			
0	2 (0.9)	1 (1.3)	12 (4.5)
1	53 (25.1)	11 (13.8)	110 (41.4)
2	56 (26.5)	31 (38.8)	46 (17.3)
3	18 (8.5)	31 (38.8)	9 (3.4)
4	3 (1.4)	6 (7.5)	0 (0.0)
Risk group (*N*, %)			
Favorable	7 (3.3)	6 (7.5)	12 (4.5)
Intermediate	115 (54.5)	48 (60.0)	163 (61.3)
Poor	44 (20.9)	25 (31.3)	55 (20.7)
Unknown	45 (21.3)	1 (1.2)	36 (13.5)
**Median OS (days), (95% CI)**			
Age			
60∼65	56 (20.8–91.2)	302 (181.9–506.9)	346 (222.8–469.2)
66∼70	67 (13.8–120.2)	129 (73.3–184.3)	316 (209.1–422.9)
71∼75	116 (44.2–187.8)	322 (136.4–507.6)	332 (138.9–525.1)
76∼	83 (35.5–130.5)	179 (8.5–349.9)	78 (0–610.4)
Performance (median, 95% CI)			
0	7	N/A	308 (0–697.8)
1	116 (48.7–183.3)	487 (0–1086.4)	427 (310.9–543.1)
2	85 (4.7–165.3)	302 (166.0–453.8)	332 (196.1–467.9)
3	62 (20.6–103.4)	232 (66.6–398.2)	22 (0–63.6)
4	18	25 (5.0–45.4)	N/A
Risk group (*N*, %)			
Favorable	190 (27.8–352.2)	347.2 (319.2–375.2)	N/A
Intermediate	101 (48.4–153.6)	380.8 (243.6–518.0)	392 (297.6–486.4)
Poor	45 (23.9–66.1)	154 (39.2–268.8)	239 (118.2–359.8)
Overall	86 (54–118)	285.6 (140–431.2)	339 (275–403)

OS, overall survival; N/A, not available; CI, confidence interval.

We could find a couple of interesting findings out of the comparison of the DACO-016 trial and the current study (Table 4). While the median age and the distribution of risk group of the patients were similar, the DACO-016 trial had more patients with better performance, as they did not recruited patients with ECOG-PS 3 or 4. However, the median OS was longer in our cohort (10.2 months *vs.* 7.7 months). And again, when we compare these two data at the same ECOG-PS or risk groups, it seems that outcomes of our cohort is better, which may suggest ethnic difference of efficacy of decitabine treatment. In fact, in the DACO-016 trial, for a small subset of Asian patients who were allocated to decitabine arm (*N* = 27), the OS was 9.3 months which was the best among the whole regional subgroups (North America 6.0, Eastern Europe 6.7, and Western Europe 9.0 months). Future analysis will be needed to determine how racial differences affect outcomes of decitabine treatment.

**Table 4:** Comparison of the DACO-016 study and the current study

Demographics	DACO-016 (decitabine arm)	The current study
Age (median, range)	73 (64–89)	74 (64–86)
Performance (*N*, %)		
0∼1	184 (76.0)	12 (15.1)
2	58 (24.0)	31 (38.8)
3	Not recruited	31 (38.8)
4	Not recruited	6 (7.5)
Risk group (*N*, %)		
Favorable	Not recruited	6 (7.5)
Intermediate	152 (63.1)	48 (60.0)
Poor	87 (36.1)	25 (31.3)
**Median OS (months) (95% CI)**		
Performance		
0∼1	8.6	11.5 (6.6–16.4)
2	5.3	
3∼4	Not recruited	4.4 (2.4–6.4)
Risk group		
Favorable	Not recruited	12.4 (11.4–13.4)
Intermediate	9.4	13.6 (8.7–18.5)
Poor	5.7	5.5 (1.4–9.6)
Overall	7.7 (6.2–9.2)	10.2 (5.0–15.4)

The current study has several limitations. Data were collected in a retrospective manner and from multiple centers. However, as we assessed OS, we believe the validity of the conclusive data and rather, it may reflect real world clinical outcomes. In addition, we could not provide the data for subsequent treatment after decitabine failure. Even if there is no known effective treatment after decitabine failure, overall survival could have been affected by the subsequent therapy. And the subsequent bone marrow examinations were performed in only 35 (43.8%) patients which may hinder to assess response rate. As the patients were old and in poor performance status, it was difficult to perform subsequent bone marrow examinations. In fact, in the DACO-016 trial, out of 485 patients, 132 patients (27.2%) did not undergo bone marrow evaluation. And in the real world situation, as there are no effective second-line treatment options for these patients, clinicians may be reluctant to perform bone marrow examinations. And, as we focused on the efficacy of the treatment, the current study does not provide detailed safety profiles other than infectious events.

## CONCLUSIONS

While the treatment options for elder AML patients have been limited, our real world data suggest that decitabine could be an effective treatment of choice, especially in Korean patients.
